# When the theory of mind would be very useful

**DOI:** 10.3389/fpsyg.2015.01449

**Published:** 2015-09-29

**Authors:** Piergiorgio Battistelli, Alessandra Farneti

**Affiliations:** ^1^Department of Psychology, University of BolognaBologna, Italy; ^2^Faculty of Education, Free University of Bozen-BolzanoBolzano, Italy

**Keywords:** theory of mind, meta-representative thought, metacognition, attributions, naïve, realism, ignorance

## Introduction

The theory of mind is certainly necessary. The dire consequences of a missing theory of mind in autism spectrum disorders are a clear proof for it. So, it might be interesting to examine how it is necessary and useful by looking at some specific (and possible) functions in the mind of a child as well as of an adult, beyond the more general comprehension of one's own and others' mental state.

First of all, as the field of the theory of mind is full of different approaches, we want to specify, very schematically, our point of view about this topic.

1. The theory (naïve) of mind is based upon the capacity to think about thoughts as such, to represent the representations regardless of the “objective” reality (meta-representative thought). The analysis of the results of the common cognitive test for studying this topic, the famous *false belief task*, shows that, regarding the representations of others, a correct meta-representation has to desist from reality as well as from one's own representation: “I know X, but I understand that you know Y.” In our opinion, the meta-representative thought is the cognitive process that generates the decentralized thought (vs. the egocentric thought); in other words the ability to adopt the cognitive perspective of someone else. However, the meta-representative thought can be pointed not only towards the external world and to other people, constituting the basis of interpersonal relations, but also towards the internal world, i.e., to one's own representations, thus constituting the basis of consciousness and meta-cognition. Summarizing, one could say that the theory of mind is the basis for the representation of the world of subjectivity (we have exposed these concepts more broadly in Battacchi et al., 1998).
2. When talking about everyday thinking, we must remember that people not always reason according to the formal logic, but often activate cognitive processes that are less rational and affected by important biases (heuristics, analogical thinking, etc…). We can apply this well-known principle also to the meta-representative thought. This is certainly a complex thought that we would be able to use, but that we don't necessarily use in every situation of daily life. Indeed it's easy to notice (and even to demonstrate, as had been done in some cases, Keisar et al., 2003; Bloom and Birch, 2007; Keisar, 2007; Ryskin and Brown-Schmidt, 2014) that in daily life people very often violate the properties of the meta-representative thought, as the ones that we have indicated above.

Massaro et al. (2013), for example, investigated the so-called outcome bias and hindsight bias in primary school children and explored the possible predictive function of false belief understanding in reducing these biases.

This is why our basic question should sometimes be: “*How useful would the theory of mind be?*” From this point of view, we believe it could be interesting to address two specific topics: one is what we will call the “*naïve realism*,” the other is “ignorance,” once more of one's own as well as of others. We belief in fact that the theory of mind, as meta-representative thought, can contribute to reduce the bias of naïve realism and to increase the meta-cognitive awareness of one's own and others' ignorance and that these functions can have relevant social implications.

## The naïve realism

Back in 1926, J. Piaget described in a very short passage the meaning of his first, by now almost forgotten studies about infantile representations. When representing the world, a thought (not only of children) can be objective or realistic. It is objective when “*it recognizes (…) the manifold intrusions of the ego into our everyday thoughts and the thousands of illusions that derive from it—illusions of senses, of language, of points of view, of values, etc.—which start to get rid of the obstacles of the ego before venturing a guess*.” On the other hand, a realistic way of thinking “*consists in ignoring the presence of the ego, which is in considering one's own perspective as immediately objective and absolute. Therefore, realism is (…) all the countless illusions that pervade the history of science*.”

The most famous Italian novel of the nineteenth century, *I promessi sposi* (“The Betrothed”) by Alessandro Manzoni, contains a very effective literary image of this concept. Manzoni tells the story of plague that haunted the city of Milan in 1630: back then, it was assumed that the epidemic had been caused by some suspicious characters, the so-called *greasers*, who greased walls and doors with a deadly substance. Renzo, the main character of the novel, is mistaken for a *greaser* because of some of his actions; he gets denounced and chased by the bulk. Manzoni then tells us that in later years people started to contest this myth of the *greasers*, and that the passer-by who had denounced Renzo argued that “*one must have seen things*.” By using the verb “to see,” Manzoni, a very subtle psychologist *ante litteram*, wants to exclude the fact that in the mind of that alas nameless person there wasn't even the slightest doubt about his representations as well as the slightest awareness of the “*manifold intrusions of the ego into everyday thinking*,” as said Piaget (1926).

It seems obvious that these words can by referred to extremely broad fields of knowledge: from the problems of micro- and macro-social relations (which are right now in some way particularly relevant) to the problems of epistemology (naive as well as scientific). Moreover, the awareness of one's subjectivity is always essential for recognizing the subjectivity of others.

This topic can also be connected to another classical theory of modern psychology: the theory of attribution (Heider, 1958). One of the most important cognitive procedures is without doubt the search for the causes of events and behaviors. We know that we can ascribe the actions of someone (and also of ourselves) to internal causes (abilities, motivations, etc.) as well as to external causes (the characteristics of objects, the behaviors of others, the chance, etc.). We know as well that there is a general tendency to overestimate the internal causes, to see ourselves as governed by ourselves or to prefer internal or external attributions as a means of valorizing and defending the image of ourselves. Today, we have the instruments for measuring the attributional processes and for documenting their importance for a person's adaptation and wellbeing. The most famous example is certainly the concept of *locus of control*. Less known and less documented, but nevertheless highly plausible is the hypothesis of a tendency to belief realistic the representations which regulate the more or less problematic relationships with *the other, the different one*, etc. A slightly more sophisticated meta-representative thought would allow us to get aware of the fact that some of our trusted representations (that seem to be completely “objective”) are nothing else than the result of our “attributions,” based on certain heuristics well known in social psychology. This is why we can ask ourselves if a metacognitive consciousness of the internal components of our representations could be useful to control the social stereotypes which are the basis of the hostile relationships typical of our societies.

## The representation of ignorance

In our opinion, there is another important topic related to meta-representation which does not get the attention it deserves, maybe because it is a kind of “non-representation.” We are referring to “ignorance,” i.e., absence of knowledge. Ignorance is a cognitive condition that we experience daily and that we consider as nothing more than a limit that has to be eliminated. If there is something we do not know, we look for information; or if there is someone who doesn't know something, we try to give him the necessary information. But reality is not as simple. If ignorance itself can be seen as something negative, the metacognitive awareness of ignorance can, on the contrary, be considered as an important and very “useful” condition. The philosopher Nicholas of Cusano (1440) referred to this in his famous oxymoron “de docta ignorantia”: in front of the immense wisdom of God, we become aware of the limits of our thoughts. In an attempt to secularize this concept, we could say that the awareness of the limits of our knowledge would be a sign of great intellectual (as well as academic) virtue—just in the sense of Socrates and his “knowing to know nothing.” On the contrary, the illusion of knowing everything is the royal road to ignorance and stupidity.

## Our ignorance

Even in a metacognitive sense, the awareness of one's ignorance is the necessary condition for every kind of learning (Rohwer et al., 2012). When do we “decide” that we know enough so that we can stop asking and learning? Which is the level of comprehension that we consider as sufficient and that leads us to stop searching? It is obvious that even the most simple of topics can never be studied and known in all of its details. Our meta-decisions depend on our meta-representation of our relative ignorance, as well as on other factors, first of all the necessity of daily routines, but also the image of ourselves, the comparison with others, etc. We can also assume that people differ in their level of tolerated ignorance: there are those who content themselves with fairly superficial skills and explanations in the illusion to eliminate their ignorance as easily as possible, and there are those who use their ignorance as a means to go on looking for a deeper knowledge. And it is exactly in this, until now only partially explored research area that we see a big potential for new studies connected with the topic of meta-representative thought.

## The ignorance of others

The ignorance of others is part of our everyday life: when we communicate something to someone, we do this mostly because we assume that the other does not know it yet and that we have to consider, in an implicit and maybe unconscious way, his or her ignorance. This means that we are capable of representing it to ourselves. Besides, ignorance is also the specific object of numerous important professions: teachers, journalists, technicians (when they must sharing their knowledge with the novice), etc. The main problem of these professions is a correct representation of ignorance: what is the state of mind of the person who ignores what I have to teach him? What does he know, and what does he not know? What are his cognitive abilities, and which ones does he not have? Representing the mental representation of a person who does not know something is probably one of the most difficult cognitive operations at all. It is all about taking on the perspective of someone else with regard to a topic that we know well, and the first, necessary but not sufficient step is that of ignoring what we know. This is certainly the main difficulty of teaching in every form and at every level. What does a child know about the world that we are supposed to explain? What are his strange representations of it? Many years ago, a boy attending the first year of junior high school, a really good student, could not understand how a river can flow northward; the only way to explain it was to take down the map from the wall, to spread it out on the floor and to allow the boy to walk on it.

It might interesting to remember that Piaget based his research method on the analysis of mistakes.

Are the mistakes of our students false representations that one has to get rid of as soon as possible, or are they a precious source for understanding their relative “ignorance”?

Road signs are another funny example: when a passer-by asks for the right direction, giving him a useful answer implies that we identify ourselves with his ignorance—something that those who are in charge of road signs are not always capable of doing.

Our everyday experience shows that explaining the functioning and use of an instrument to a non- professional is a very difficult task for technicians in every field—we see this by the countless obscure user manuals (Serra Barneto, 1992). Even in this not all too trivial field, meta-representation could be very useful for keeping in mind the ignorance of others.

In conclusion, we can briefly highlight that the theory of mind and the meta-cognition are not only important mechanisms of information processing but they represent overall the essential tools of every kind of interpersonal communication.

## Conflict of interest statement

The authors declare that the research was conducted in the absence of any commercial or financial relationships that could be construed as a potential conflict of interest.
